# Anencephaly: a rare clinical image

**DOI:** 10.11604/pamj.2022.42.11.35107

**Published:** 2022-05-06

**Authors:** Mayur Bhaskar Wanjari, Tejaswee Lohakare

**Affiliations:** 1 Department of Research and Development, Jawaharlal Nehru Medical College, Datta Meghe Institute of Medical Sciences, Sawangi, Wardha, Maharashtra, India,; 2 Department of Child Health Nursing, Smt. Radhikabai Meghe Memorial College of Nursing, Datta Meghe Institute of Medical Sciences, Sawangi, Wardha, Maharashtra, India

**Keywords:** Anencephaly, protruded eyeballs, ultrasound sonography test

## Image in medicine

Anencephaly is a lethal fetal neurological malformation. This malformation accounts for 40% of neural tube malformations. The diagnosis is based on the ultrasound of the first trimester between the 11^th^ and the 14^th^ weeks of amenorrhea by discovering an exencephaly which results in the visualisation of the ossification of the cranial box and, therefore, the impossibility of measuring the biparietal diameter. Here we present the case of anencephaly; a female patient comes to the gynaecology department for the delivery on the ultrasound sonography test (USG) examination to know the health status of the fetus show anencephaly. On the second day, patient delivery was done, and the patient´s newborn child was seen with low set ears and protruded eyeballs.

**Figure 1:**
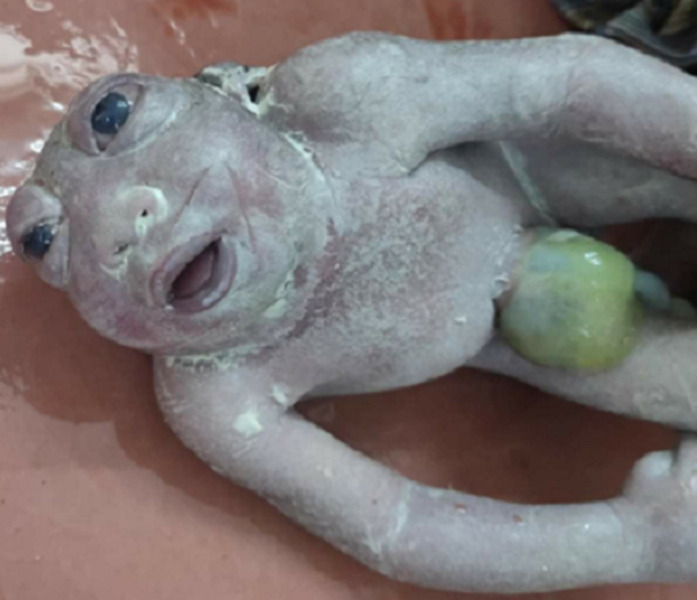
newborn with anencephaly, low set ears, and protruded eyeball

